# Multiline treatment of primary cutaneous adenoid cystic carcinoma with multiple metastases: a case report and literature review

**DOI:** 10.3389/fonc.2025.1669171

**Published:** 2025-11-25

**Authors:** Xiaotao Geng, Daqing Sun, Huimin Sun, Chunyan Zhang, Jianwen Li, Furong Hao, Xiaolong Chang

**Affiliations:** 1 Department of Radiation Oncology, Weifang People’s Hospital, Shandong Second Medical University, Weifang, China; 2 Department of Pathology, Weifang People’s Hospital, Shandong Second Medical University, Weifang, China; 3 Radiation Oncology, Anqiu People’s Hospital, Weifang, China

**Keywords:** primary cutaneous adenoid cystic carcinoma, surgery, radiotherapy, chemotherapy, immunotherapy, PD-1 inhibitor

## Abstract

**Background:**

Primary cutaneous adenoid cystic carcinoma (PCACC) is an extremely rare malignancy, typically presenting as a slow-growing, painless cutaneous mass with a relatively favorable prognosis compared to adenoid cystic carcinomas (ACC) of salivary gland origin. Metastatic PCACC is uncommon, with stage IV disease accounting for approximately 4% of reported cases. Due to its rarity, clinical understanding of the disease course and optimal management strategies remains limited.

**Case presentation:**

We report the case of a 57-year-old male who presented with a painful ulcerated mass on the left ankle. Magnetic resonance imaging (MRI) revealed a subcutaneous lesion, and histopathological examination following surgical excision confirmed the diagnosis of ACC. Immunohistochemistry was consistent with ACC, and a PET-CT scan excluded regional or distant disease, supporting the diagnosis of PCACC. The patient subsequently underwent adjuvant radiotherapy. However, within eight months, follow-up imaging revealed pulmonary and osseous metastases. A biopsy confirmed metastatic ACC consistent with the primary lesion. The patient was treated sequentially with three lines of systemic therapy, including chemotherapy, immunotherapy, and targeted therapy, along with palliative radiotherapy. Despite temporary disease stabilization, the malignancy progressed rapidly, with new pulmonary, intracranial, and extensive skeletal metastases. The patient eventually discontinued treatment and was lost to follow-up.

**Conclusion:**

This case highlights an unusually aggressive clinical course of PCACC with rapid progression to widespread metastatic disease shortly after definitive local treatment. Given the rarity of metastatic PCACC, this report contributes valuable insight into its natural history, diagnostic approach, and therapeutic challenges. It underscores the need for heightened clinical awareness and further research into effective systemic treatment strategies for advanced PCACC.

## 1 Introduction

Adenoid cystic carcinoma (ACC) is a rare histological subtype that most commonly arises in the major and minor salivary glands but can also occur in other anatomical sites, including the breast, trachea, lungs, cervix, lacrimal glands, and skin (1). ACC arising in the skin, in the absence of involvement of other body sites, is defined as primary cutaneous adenoid cystic carcinoma (PCACC) (2). PCACC is exceedingly rare and has been reported predominantly through individual case reports. It can occur in various anatomical regions, such as the scalp, face, chest, back, and upper and lower extremities, typically presenting as a painless mass. Compared to ACC of the major salivary glands, PCACC has been associated with a relatively favorable prognosis (1). Furthermore, population-based data from the SEER database indicate that stage IV, or metastatic, PCACC represents only approximately 4% of all reported cases (3). In this report, we describe a rare case of PCACC originating in the left ankle that exhibited rapid progression to metastatic disease shortly after initial treatment. We provide a comprehensive account of the patient’s clinical course, including surgical resection, postoperative radiotherapy, and subsequent systemic therapies.

## 2 Case presentation

**Figure 1:**
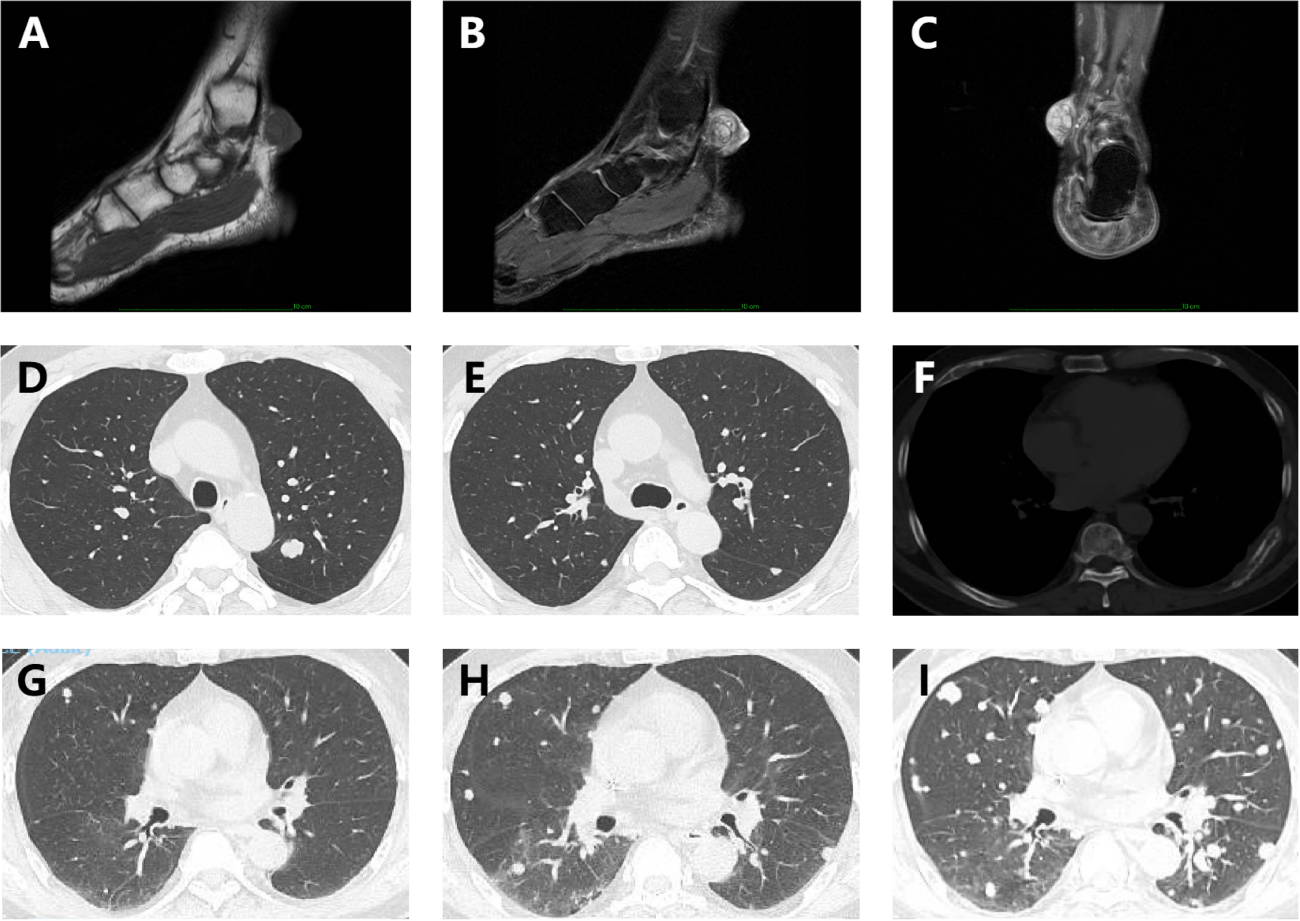
Initial MRI of the left ankle at disease onset and serial follow-up chest CT images. **(A-C)** Magnetic Resonance Imaging (MRI) revealed a well-defined, round mass located within the subcutaneous fat layer of the medial aspect of the left posterior ankle. On sagittal T1-weighted imaging, the lesion appears isointense; On sagittal and coronal fat-suppressed T2-weighted imaging, the lesion demonstrates heterogeneous hyperintensity with internal linear hypointense strands. **(D-F)** Chest CT performed on August 3, 2021, demonstrated multiple metastatic lesions in both lungs as well as a metastasis in the left eighth rib. **(G)** Chest CT on January 10, 2022; **(H)** Chest CT on March 9, 2022; **(I)** Chest CT on May 26, 2022.

**Figure 2:**
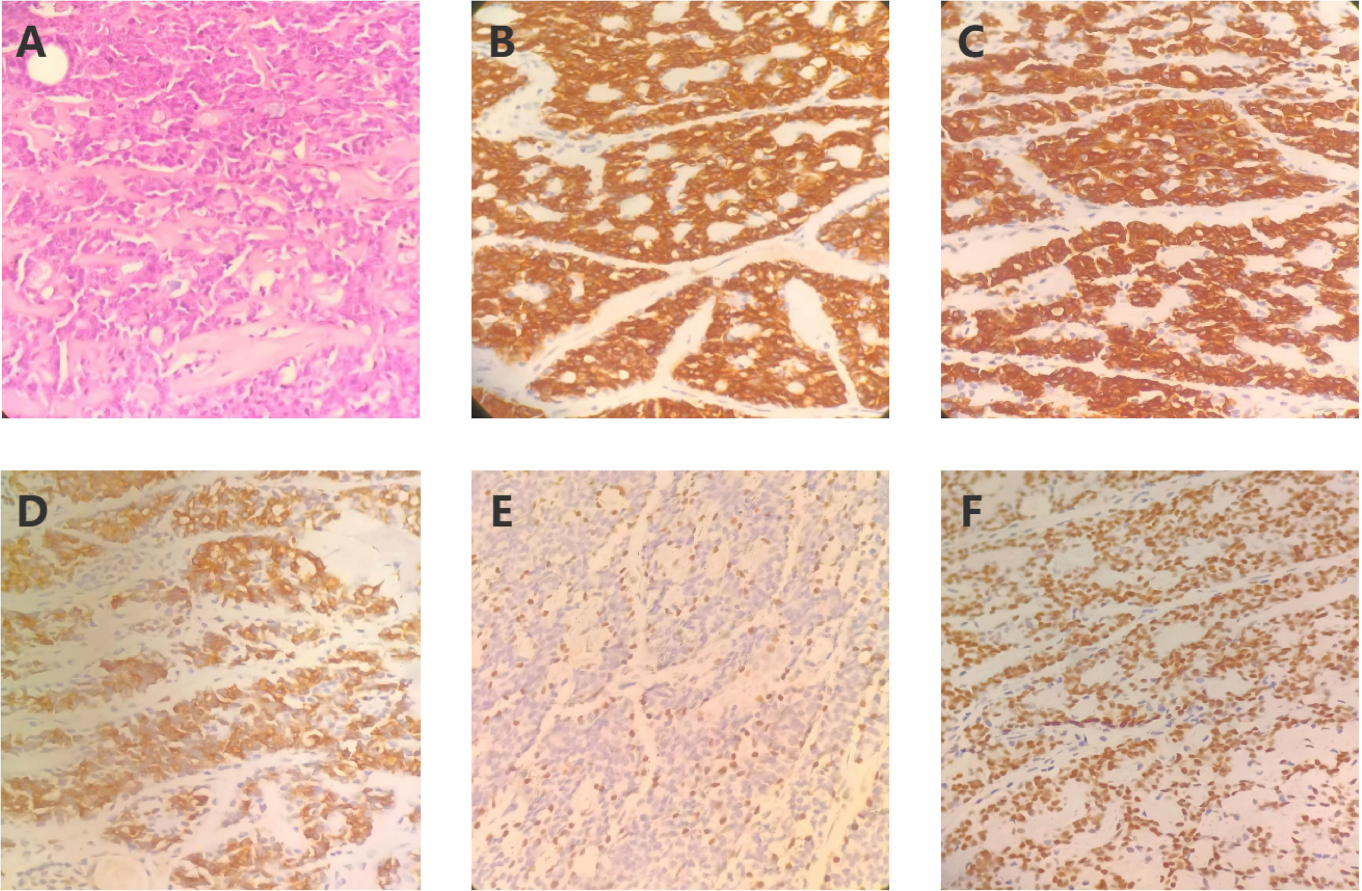
Histopathological findings of the resected left ankle tumor. **(A)** ematoxylin and eosin (H&E) staining of the primary tumor (original magnification ×400); **(B)** Immunohistochemical staining showing CD117 positivity (original magnification ×400); **(C)** IHC showing CK7 positivity (original magnification ×400); **(D)** IHC showing CK8/18 positivity (original magnification ×400); **(E)** IHC showing P63 positivity (original magnification ×400); **(F)** IHC showing SOX-10 positivity (original magnification ×400).

A 57-year-old middle-aged male presented to a local hospital on December 10, 2020, with a painful skin mass on the left ankle, accompanied by localized ulceration. Magnetic Resonance Imaging (MRI) revealed a well-defined, round mass located within the subcutaneous fat layer of the medial aspect of the left posterior ankle. The lesion appeared isointense on T1-weighted imaging (T1WI) and demonstrated heterogeneous hyperintensity with internal linear hypointense strands on fat-suppressed T2-weighted imaging (T2WI) (**Figure 1**). Then the patient underwent surgical excision on December 15, 2020. Postoperative pathology confirmed adenoid cystic carcinoma (ACC) (**Figure 2A**), with no tumor involvement at the peripheral or deep margins. Immunohistochemical (IHC) analysis demonstrated positivity for CD117, CK7, CK8/18, P63, and SOX-10 (**Figures 2B-F**). The patient was referred to our institution for adjuvant radiotherapy. A PET-CT was performed to exclude residual disease in the surgical bed and distant metastases. From January 8, 2021, to March 3, 2021, the patient received postoperative adjuvant radiotherapy. The gross tumor volume (GTV) of tumor bed (GTVtb) encompassed the post-surgical tumor bed area of the left ankle. A certain margin around the GTVtb was expanded to define the clinical target volume (CTV). The prescribed radiation doses were 64 Gy and 54 Gy in 30 fractions for the planning target volumes (PTVs) derived from the GTV and CTV, respectively.

**Figure 3:**
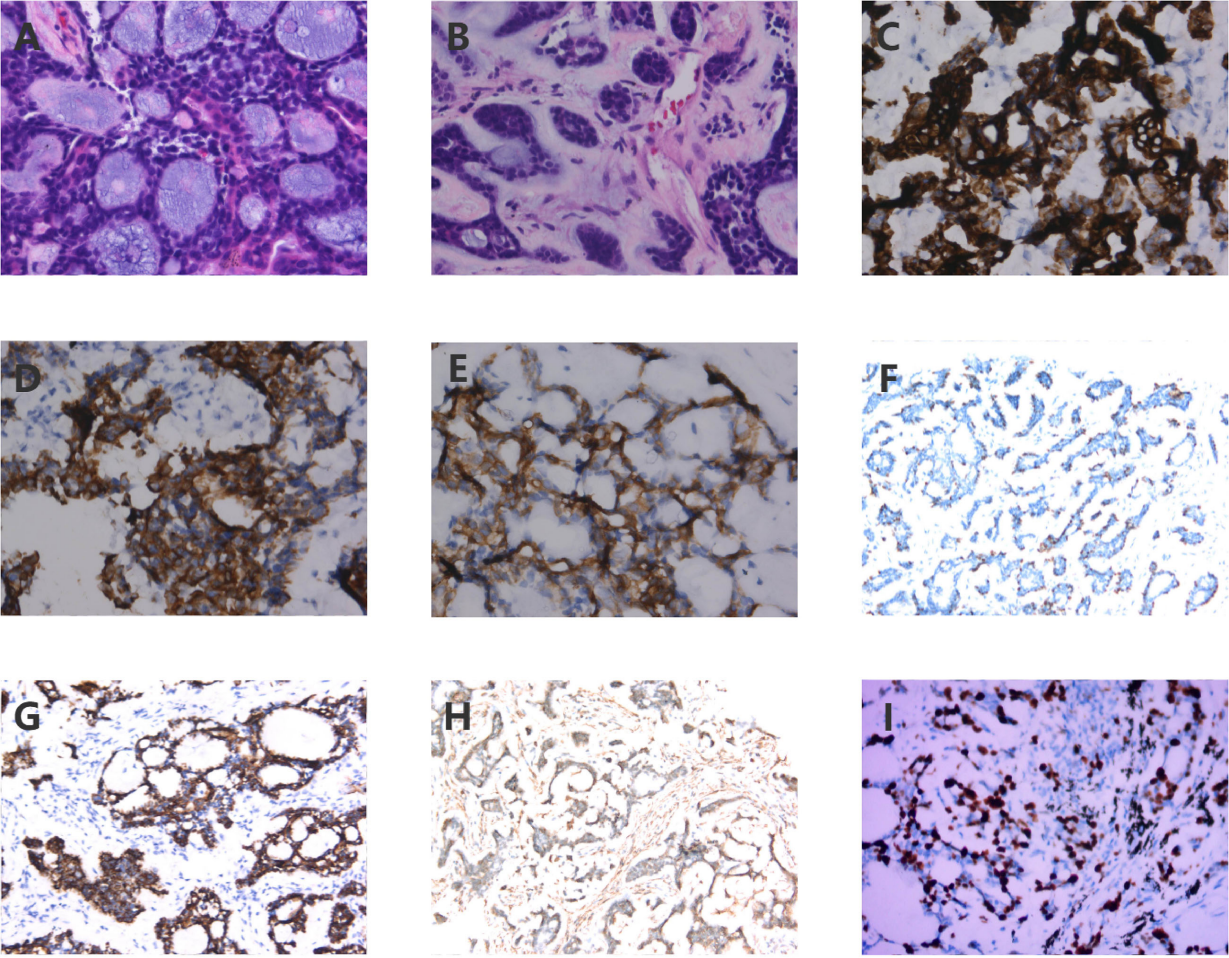
Histopathology and immunohistochemistry **(IHC)** of the pulmonary metastatic lesion. **(A)** Cribriform architecture in the pulmonary metastatic lesion: Most tumor cells are arranged in well-defined pseudoglandular cribriform structures, which appear round or oval in shape. Basophilic mucinous material is visible within the lumina (original magnification ×400); **(B)** Tubular architecture in the pulmonary metastatic lesion: In some areas, tumor cells are arranged in tubular gland-like structures, with an inner layer of epithelial cells and an outer layer of myoepithelial cells(original magnification ×400). **(C)** IHC staining showing CD117 positivity (original magnification ×400); **(D)** IHC showing CK7 positivity (original magnification ×400); **(E)** IHC showing CK8/18 positivity (original magnification ×400); **(F)** IHC showing P63 positivity (original magnification ×400); **(F)** IHC showing P63 positivity (original magnification ×400); **(G)** IHC showing CK5/6 positivity (original magnification ×400); **(H)** IHC showing SMA positivity (original magnification ×400); **(I)** IHC revealed a Ki-67 proliferation index exceeding 50%.

**Figure 4:**
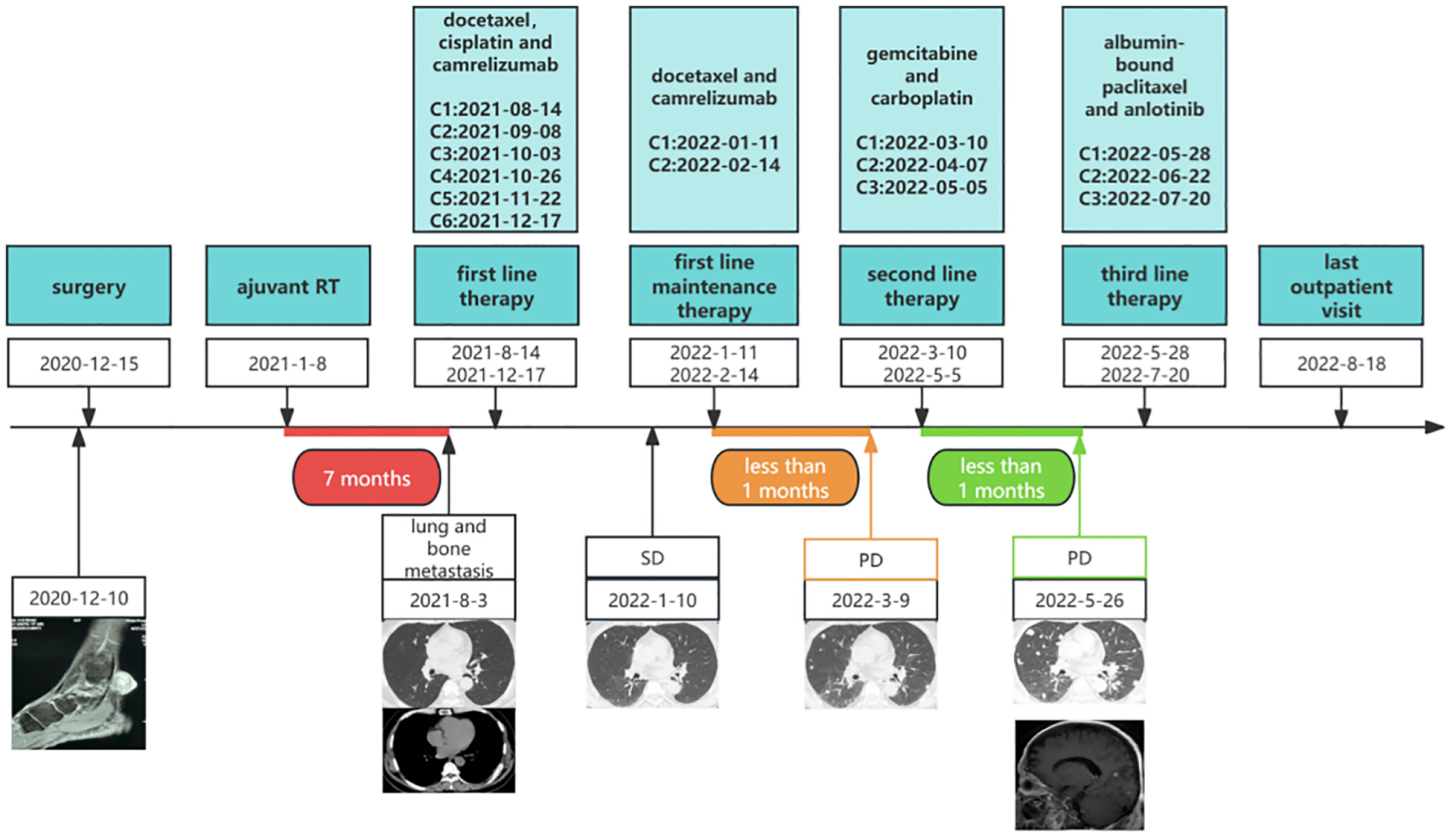
Timeline of the patient’s overall treatment course, including surgery, postoperative radiotherapy, first-line therapy, first-line maintenance therapy, second-line therapy, and third-line therapy. C, cycle; RT, radiotherapy; SD, stable disease; PD, progressive disease.

During routine follow-up on August 3, 2021, less than eight months after the initial diagnosis, multiple pulmonary metastases and a metastatic lesion in the left eighth rib were detected. (**Figures 1D-F**). The pathological biopsy of the pulmonary metastatic lesions confirmed adenoid cystic carcinoma, with the cribriform component accounting for 90% and the tubular component for 10% (**Figures 3A, B**). The mitotic count was 2–3 mitoses per high-power field (HPF). IHC staining was positive for CD117, CK7, CK8/18 (epithelial component), P63, CK5/6, SMA (myoepithelial markers), and negative for S-100, TTF-1, Napsin A, CD56, and Synaptophysin. The Ki-67 proliferation index was approximately 50% (**Figures 3C-I**). A diagnosis of PCACC with multiple pulmonary and osseous metastases was established. The patient began systemic therapy under the care of our Department of Medical Oncology. First-line treatment commenced on August 14, 2021, with the regimen consisting of docetaxel 140 mg (day 1), cisplatin 140 mg (day 1), and camrelizumab 200 mg (day 1) every 3 weeks. After six cycles, the disease was evaluated as stable disease (SD) with partial tumor shrinkage. Two cycles of maintenance therapy were initiated on January 11, 2022, using docetaxel 140 mg (day 1) and camrelizumab 200 mg (day 2) every 3 weeks. On March 9, 2022, a follow-up chest CT revealed new pulmonary lesions, indicating disease progression (**Figure 1H**). Second-line therapy was initiated on March 10, 2022, with gemcitabine 1.8 g (days 1 and 8) and carboplatin 0.5 g (day 2) every 3 weeks. During this period, from March 23 to April 22, 2022, the patient received palliative radiotherapy for the left rib metastasis at a local hospital, with a total dose of 39 Gy administered in 13 fractions (3 Gy per fraction). On May 5, 2022, the patient received the third cycle of second-line therapy. Unfortunately, CT on May 26, 2022, showed newly developed pulmonary lesions (**Figure 1I**), and concurrent brain MRI identified multiple brain metastases, indicating further progression. Third-line therapy was initiated on May 28, 2022, comprising albumin-bound paclitaxel 400 mg (day 1) and anlotinib 12 mg (days 1–14) every 3 weeks. After two cycles, MRI revealed new metastatic lesions involving T11, L1, L2, S2 vertebrae, as well as the right iliac bone, ischium, and femur, indicating further disease progression. Although palliative radiotherapy for bone metastases was planned, the patient declined further treatment and was subsequently lost to follow-up. The timeline of the patient’s treatment, including surgery, postoperative radiotherapy, and subsequent systemic therapy following disease progression, is presented in **Figure 4**.

## 3 Discussion

Adenoid cystic carcinoma can arise in various locations, including the major salivary glands, minor salivary glands, breast, skin, lungs, trachea, and the female reproductive system. When diagnosing primary cutaneous adenoid cystic carcinoma, it is important to carefully distinguish it from metastatic adenoid cystic carcinoma of salivary gland origin and from other cutaneous adnexal tumors. Metastatic adenoid cystic carcinoma of salivary gland origin indicates the presence of a primary tumor in the salivary glands and can present in two distinct scenarios. In cases of synchronous metastasis, the primary salivary gland lesion can be identified through PET-CT, CT, MRI, ultrasonography, and confirmatory biopsy. For metachronous metastasis, it is essential to inquire about any prior history of primary salivary gland adenoid cystic carcinoma. The cutaneous adnexal tumors that require differential diagnosis include sebaceous carcinoma, endocrine mucin-producing sweat gland carcinoma, microcystic adnexal carcinoma, and porocarcinoma. Histologically, sebaceous carcinoma is characterized by a lobular or sheet-like architecture with variable degrees of cellular differentiation, often displaying foamy cytoplasm and areas of necrosis. Immunohistochemically, it typically shows positive expression of epithelial membrane antigen (EMA), androgen receptor (AR), AE1/AE3, and adipophilin (4). Endocrine mucin-producing sweat gland carcinoma is a rare, low-grade malignancy predominantly found in elderly women, often occurring in the eyelids. Clinically, it presents as a slow-growing, painless nodule. Histologically, the tumor has well-defined borders, and the cells are arranged in solid nests, papillary, or sieve-like structures. Mucin is prominently present in the cytoplasm, and in some cases, there is a coexisting invasive mucinous carcinoma component. Immunohistochemically, the tumor cells are positive for cytokeratin 7 (CK7), epithelial membrane antigen (EMA), gross cystic disease fluid protein 15 (GCDFP-15), GATA binding protein 3 (GATA3), synaptophysin, chromogranin, neural cell adhesion molecule (CD56), and neuron-specific enolase (NSE), suggesting biphasic differentiation toward both apocrine sweat glands and neuroendocrine cells (5). Microcystic adnexal carcinoma is most commonly found in middle-aged to elderly Caucasians, typically occurring in the midline region of the face. It presents as a slowly enlarging, firm nodule or plaque that is skin-colored or pale yellow, often associated with sensory abnormalities, suggesting nerve involvement. Histologically, the superficial layers show keratinized microcysts and ductal structures, while the deeper layers display dense, cord-like cellular infiltration with hyaline, glassy stroma surrounding nerves. The cellular morphology is relatively mild, but the infiltration is extensive. While lacking a specific immunohistochemical signature, positivity for carcinoembryonic antigen (CEA), epithelial membrane antigen (EMA) and cytokeratin 7 (CK7) combined with negative Ber-EP4 staining is helpful in differential diagnosis (6). Porocarcinoma is most commonly seen in elderly individuals, typically occurring on the head, neck, or lower extremities. It presents as a slowly enlarging, firm nodule or ulcerated plaque, often accompanied by pain or pruritus. Histologically, the tumor consists of atypical poroid-like basal cells arranged in solid nests, with mature ducts, comedone-like necrosis, and squamous differentiation frequently observed. The infiltration pattern may be pushing, infiltrative, or Paget-like. Positive immunohistochemical staining for carcinoembryonic antigen (CEA), epithelial membrane antigen (EMA), cytokeratin 19 (CK19), CD117 (c-kit), and Ber-EP4 aids in establishing the diagnosis. Abnormal expression of p53, retinoblastoma protein (Rb), p16, and nuclear protein in testis (NUT) suggests malignant characteristics (7).

Given the diagnostic complexities and histological mimics outlined above, it is important to recognize not only the primary tumor characteristics but also the clinical behavior and metastatic potential of primary cutaneous adenoid cystic carcinoma (PCACC). PCACC is relatively rare and is mostly reported in the form of individual case reports or case series. Metastatic PCACC is even rarer. We conducted a search and summarized the reports of metastatic primary cutaneous adenoid cystic carcinoma from the PubMed database (**Table 1**) (8–28). A total of 23 cases of metastatic PCACC were included in the literature review, comprising 10 male and 13 female patients. The age of onset ranged from 37 to 83 years, with a median age of 57 years. The most common primary sites were the scalp and face, followed by the chest and back, while involvement of the limbs was less frequent. The most common metastatic sites included regional lymph nodes, lungs, and bones. Less common sites of metastasis included the brain, kidneys, and nasal septum. Eleven patients presented with synchronous metastases, while 12 had metachronous metastases. Among the metachronous cases, three developed regional lymph node metastases within two years of the initial diagnosis, while the remaining cases had a relatively long time interval between the primary tumor and distant organ metastasis, ranging from 4 to 23 years. Following metastasis, treatment was individualized based on the patient’s condition, with options including surgery, radiotherapy, and chemotherapy. Currently, there is no standard treatment protocol for adenoid cystic carcinoma. The chemotherapy regimens used in the studies we reviewed included vinorelbine and cisplatin, 5-flurouracil and cisplatin, Cyclophosphamide, doxorubicin, and cisplatin, and Adriamycin and cisplatin. In summary, the chemotherapy regimen for PCACC is typically based on platinum-based combination chemotherapy.

**Table 1:** Previously reported cases of metastatic primary cutaneous adenoid cystic carcinoma.

Year	Authors (reference)	Sex	Age	Primary site	Metastasis site	Metachronism/ simultaneity	Time interval from initial presentation to metastasis	Treatment
2025	Kelsey E (8)	F	60s	left thigh	kidney, bone	metachronism	17 years(kidney) 18 years(bone)	surgery+ palliative radiotherapy
2024	El Mansoury FZ (9)	M	79	hand	lung	simultaneity	/	Chemotherapy (VC)
2023	Dantis K (10)	F	65	right posterior chest wall	axillary lymph node	simultaneity	/	surgery
2022	Takada Y (11)	F	59	left mammary papilla	nasal septum, lung	metachronism	5 years(left lung lesion) 13 years(right lung lesion) 17 years(nasal septum)	surgery
2022	Lv JJ (12)	M	54	left forehead	brain	metachronism	103 months	surgery
		F	53	scalp	lung, bone and shoulder	metachronism	54 months	radiotherapy+ chemotherapy
		F	49	scalp	lung and pleura	simultaneity	/	chemotherapy
2021	Sato Y (13)	M	70	right upper back	axillary lymph node	simultaneity	/	surgery
2019	Takegawa M (14)	M	83	left lower leg	inguinal lymph node	simultaneity	/	surgery
2017	Singh GK (15)	M	57	right nasolabial fold	lung	simultaneity	/	palliative radiotherapy+ chemotherapy (CF)
2017	Prieto-Granada CN (16)	M	81	right tibial	inguinal lymph node	simultaneity	/	surgery+ adjuvant radiotherapy
2016	Mohamed Ali AA (17)	F	65	left temporal region pos- terior to the pinna	neck lymph node, lung and bone	simultaneity	/	radiotherapy+ chemotherapy
2016	Pozzobon LD (18)	M	67	first metacarpal	axillary lymph node, lung and brain	metachronism	2 years(axillary lymph node) 4 years(lung) 5 years(brain)	radiotherapy+ chemotherapy (CAP)+ surgery
2016	Pohl U (19)	F	57	left groin	lung, chest wall, hilar lymphadenopathy and brain	metachronism	13 years(lung, chest wall, hilar lymph node) 17 years(brain)	palliative radiotherapy+ surgery
2014	Rocas D (20)	F	47	upper outer quadrant of the left breast	axillary lymph node	metachronism	18 months	surgery+ adjuvant radiotherapy
2010	Singh A (21)	M	55	infranasal area above the lip	lung	simultaneity	/	Chemotherapy (CF)
2004	Doganay L (22)	F	55	left axilla	axillary lymph node and lung	metachronism	2 years	surgery
2001	Chu SS (23)	M	64	left parotid region	neck lymph node	simultaneity	/	surgery+ adjuvant radiotherapy
2000	Weekly M (24)	M	40	scalp	neck lymph node	simultaneity	/	surgery+ adjuvant radiotherapy
1998	Zimmerman RL (25)	F	82	scalp	lung	metachronism	10 years	NM
1989	Ikegawa S (26)	F	37	the frontal region	lung	metachronism	23 years	Chemotherapy (AP)
1987	Seab JA (27)	F	48	scalp	lung and pleura	metachronism	7 years	NM
1975	Sanderson KV (28)	F	57	scalp	lung	metachronism	10 years	surgery

F, female; M, male; NM, not mentioned; VC, vinorelbine and cisplatin; CF, cisplatin and 5-flurouracil; CAP, Cyclophosphamide, doxorubicin, and cisplatin; AP, Adriamycin and cisplatin.

Previous studies have suggested that ACC is a highly aggressive tumor with indolent growth and a tendency for delayed metastasis. The studies we reviewed also supported this notion. However, the case we report deviates from this characteristic, as the patient developed multiple bilateral lung metastases and bone metastasis just 8 months after the initial diagnosis. Previous research has confirmed that tumors with a predominance of solid components or those associated with high-grade epithelial transformation are more invasive and have a poorer prognosis (29). To investigate the rapid progression in our case, we revisited the pathology of the pulmonary metastatic lesions and found that they were predominantly cribriform in structure, with a small amount of tubular components (90% cribriform + 10% tubular). No solid components or high-grade transformation were observed. Therefore, it is unfortunate that the cause of the rapid disease progression in this case remains unclear. The patient received first-line treatment with docetaxel, cisplatin, and camrelizumab (a programmed cell death protein 1 inhibitor), followed by first-line maintenance therapy with docetaxel and camrelizumab. However, the disease progressed rapidly thereafter. Second-line treatment with gemcitabine and carboplatin, and third-line treatment with albumin-bound paclitaxel combined with anlotinib (an anti-angiogenesis agent), did not yield the desired efficacy. Systemic treatment strategies for metastatic primary cutaneous ACC primarily refer to the treatment protocols for head and neck ACC. Currently, the efficacy of a combined docetaxel and cisplatin chemotherapy regimen for metastatic salivary gland adenoid cystic carcinoma is supported by two Japanese studies (30, 31). A Japanese multicenter Phase II trial investigated docetaxel plus cisplatin for recurrent or metastatic non-squamous head and neck cancer. The study enrolled 10 patients with adenoid cystic carcinoma. Within this group, three patients achieved a partial response (30). Another Japanese study demonstrated superior efficacy of docetaxel plus cisplatin over paclitaxel plus carboplatin in primary salivary gland adenoid cystic carcinoma, yielding a higher overall response rate (ORR) and longer median progression-free (mPFS) and overall survival (mOS) (31). In addition to conventional chemotherapy, recent advances in systemic treatment include immunotherapy and targeted therapy. Although we used a PD-1 inhibitor during first-line therapy and first-line maintenance, the outcome was far from optimal. We suspect that the tumor’s mutational burden and PD-L1 expression were both low. However, recent studies have also reported preliminary efficacy of immunotherapy in recurrent or metastatic ACC. A recent study on the combination of nivolumab and ipilimumab in 19 patients with recurrent or metastatic ACC demonstrated preliminary efficacy (32). The disease control rate was 42.1% (8/19), with a median overall survival of 30 months. This study provides an alternative treatment option for recurrent and metastatic ACC, beyond chemotherapy. In addition to immunotherapy, multi-target kinase inhibitors (MKIs) such as Apatinib, Lenvatinib, Axitinib, and Sorafenib have provided new hope for patients with recurrent metastatic ACC. A systematic review and meta-analysis of 28 studies on targeted therapy for recurrent metastatic ACC showed that MKIs were the most effective among all targeted categories, with better objective response rates (ORR) and progression-free survival (PFS) compared to other single-target drugs (33).

Characterized by significant heterogeneity, adenoid cystic carcinoma exhibits marked prognostic diversity among patients. This variability is largely attributable to distinct molecular subtypes, which recent studies have shown to correlate with clearly differentiated clinical outcomes. The MD Anderson Cancer Center led a study that included 54 patients with primary salivary gland adenoid cystic carcinoma. RNA sequencing and proteomic analysis were performed on all patients, and consensus clustering based on RNA-seq and RPPA data was used to identify molecular subtypes. The study classified ACC into two molecular subtypes: ACC-I and ACC-II (34). ACC-I is a high-grade, aggressive subtype driven by MYC and NOTCH signaling, characterized by a poor prognosis. In contrast, ACC-II is a more indolent subtype driven by TP63 and receptor tyrosine kinase activation (e.g., EGFR, MET) along with their downstream pathways, and is associated with a favorable long-term prognosis. A large-scale retrospective study of 438 adenoid cystic carcinoma (ACC) cases validates the prognostic and therapeutic relevance of two molecular subtypes: ACC-I, characterized by MYC overexpression and frequent NOTCH1 mutations, and ACC-II, defined by TP63 upregulation and receptor tyrosine kinase pathway activation. ACC-I tumors exhibited an immunosuppressive microenvironment, poorer response to systemic therapies, and significantly shorter overall survival compared to ACC-II (35). These findings support the integration of MYC/TP63-based classification into clinical trial design and treatment planning for ACC.

## 4 Conclusions

This case report highlights a rare instance of primary cutaneous adenoid cystic carcinoma (PCACC) originating in the left ankle, which rapidly progressed to metastatic disease shortly after initial treatment. Despite undergoing surgical resection, adjuvant radiotherapy, and multiple lines of systemic therapy, the patient experienced rapid progression of pulmonary and bone metastases. This case deviates from the typical indolent course of ACC, where metastasis tends to occur later. A thorough review of the literature reveals that metastatic PCACC is exceedingly rare, with only 23 cases reported in the PubMed database. The primary sites of PCACC most commonly involve the scalp, face, chest, and back, with metastasis typically involving regional lymph nodes, lungs, and bones. Notably, metachronous metastases can develop years after the initial diagnosis. Although treatment strategies primarily focus on surgery, radiotherapy, and chemotherapy, the lack of a standardized treatment protocol for metastatic ACC remains a challenge. In recent years, advances in immunotherapy and targeted therapy, including PD-1 inhibitors and multi-target kinase inhibitors, have shown promise in improving outcomes for metastatic ACC. However, as evidenced in this case, further investigation into the molecular characteristics and treatment strategies for rapidly progressing PCACC is essential to provide better therapeutic options for patients facing aggressive disease courses. More studies are needed to determine optimal treatment regimens and improve prognostic predictions for patients with metastatic PCACC.

## Data Availability

The datasets presented in this article are not readily available because Not applicable. Requests to access the datasets should be directed to Not applicable.
